# Spatially aware radiomics integrating anatomical knowledge to improve lymph node malignancy prediction in head and neck cancer

**DOI:** 10.1002/acm2.70483

**Published:** 2026-01-27

**Authors:** Liyuan Chen, Sepeadeh Radpour, Michael Dohopolski, David Sher, Jing Wang

**Affiliations:** ^1^ Department of Radiation Oncology UT Southwestern Medical Center Dallas Texas USA

**Keywords:** head and neck cancer, lymph node malignancy prediction, spatially aware radiomics

## Abstract

**Background:**

Radiomics holds the potential to improve the diagnostic evaluation of equivocal lymph nodes in head and neck cancer (HNC). While conventional radiomics models utilize features such as intensity, geometry, and texture of individual lymph node, they often neglect key spatial and anatomical characteristics tied to lymphatic dissemination patterns.

**Purpose:**

In this study, we propose a novel spatially aware radiomics model that integrates anatomical knowledge and clinical factors to enhance lymph node malignancy prediction.

**Methods:**

A total of 1389 lymph nodes (1119 benign and 270 malignant), contoured on CT scans from 192 HNC patients were included. Two models were developed: a baseline model using conventional radiomics features and an enhanced model incorporating five additional spatial and anatomical features, such as primary tumor type, lymph node level, the laterality of the primary tumor, the laterality of the lymph node, and the distance from the lymph node to the primary tumor. Sensitivity (SEN), specificity (SPE), accuracy (ACC), positive predictive value (PPV), negative predictive value (NPV) and the area under the receiver operating characteristic curve (AUC) criteria were used to evaluate the model performance.

**Results:**

The proposed spatially aware radiomics model significantly outperformed the baseline model. The baseline model achieved SEN = 0.915, SPE = 0.756, ACC = 0.787, PPV = 0.475, NPV = 0.974, and AUC = 0.931. The enhanced model achieved SEN = 0.919, SPE = 0.845, ACC = 0.860, PPV = 0.589, NPV = 0.977, and AUC = 0.953. Statistical testing confirmed a significant improvement in both accuracy (*p* = 3.71 × 10^−20^) and AUC (*p* = 1.13 × 10^−4^).

**Conclusions:**

This study demonstrates that incorporating lymphatic anatomy and clinical context into radiomics models significantly improves predictive performance. The proposed approach enhances interpretability, aligns with clinical workflows, and holds promises for personalized radiation therapy planning.

## 1 INTRODUCTION

Head and neck cancer (HNC) frequently metastasizes to regional lymph nodes (LNs), and accurate assessment of LN malignancy is critical for effective treatment planning. Standard imaging techniques such as computed tomography (CT) and positron emission tomography (PET) are commonly used to evaluate LN involvement. However, these methods often rely on size and morphological criteria, which can be insufficient for detecting metastases, especially in cases where malignant nodes do not exhibit significant enlargement or other distinctive features.^1^, ^2^

Radiomics has emerged as a promising approach to enhance the predictive accuracy of LN malignancy assessments. By extracting high‐dimensional quantitative features from standard imaging modalities, radiomics can capture subtle textural and spatial information that may not be apparent through visual inspection alone.^3^, ^4^ Studies have demonstrated the potential of radiomics in predicting occult LN metastases in oral squamous cell carcinoma,^5^, ^6^, ^7^ suggesting its role as a noninvasive tool for pre‐treatment evaluation. These models are typically interpretable and can be trained with relatively limited datasets—an advantage in medical imaging domains where large‐scale annotated datasets are often unavailable. ​

Parallel to radiomics, deep learning, particularly convolutional neural networks (CNNs), has emerged as a powerful tool for image‐based classification tasks. CNNs are capable of learning complex hierarchical patterns directly from imaging data without the need for handcrafted features. However, their performance is highly dependent on the size and diversity of the training dataset. In the medical domain, limited data availability and the high cost of annotation often pose significant barriers to training effective CNN models.^8^, ^9^

Importantly, both radiomics and deep learning models often treat lymph nodes as independent units, potentially overlooking contextual information that is routinely considered in clinical decision‐making. For example, the spatial relationship between a lymph node and the primary tumor, the nodal level, and laterality can all influence metastatic likelihood based on known patterns of lymphatic spread. These anatomical cues are not routinely incorporated into existing predictive models but represent valuable information for improving model specificity, particularly in clinical scenarios where overtreatment leads to increased toxicity.

This study introduces a novel, spatially‐aware radiomics model that integrates anatomical knowledge and clinical factors to improve lymph node malignancy prediction in HNC. By incorporating spatial relationships between lymph nodes and primary tumors, as well as relevant clinical features, we aim to develop a more accurate and clinically relevant predictive tool.

## 2 METHODS

### 2.1 Patient cohort and imaging data

A retrospective dataset of 192 HNC patients was collected, including CT scans with delineations of primary tumors and lymph nodes. The distribution of primary disease sites was as follows: base of tongue (*n* = 67, 34%), tonsil (*n* = 61, 32%), unspecified oropharynx (*n* = 5, 3%), larynx (*n* = 53, 28%), and hypopharynx (*n* = 6, 3%). All 192 patients were included in the final data analysis and met the inclusion criteria established below. *Inclusion Criteria*: Patients were included if they had a histologically confirmed primary HNC diagnosis (specifically, the types listed in the feature set: tonsil, base of tongue, oropharynx, larynx, or hypopharynx) and underwent preoperative contrast‐enhanced CT imaging scans, followed by surgical neck dissection. *Exclusion Criteria*: Patients were excluded if they had metastatic disease, an occult primary tumor, or if pathological status was not available or unclear.

**FIGURE 1:**
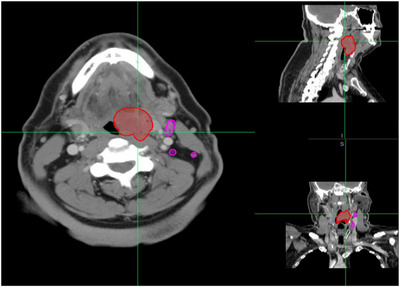
An example of the primary tumor and the lymph nodes falling in interest on a CT scan.

In total, 1389 lymph nodes were analyzed. Primary tumor and lymph node contours were initially performed by a research scientist (S.R.) and subsequently reviewed and edited by a senior radiation oncologist (D.S. with over 15 years of experience). The malignancy/benign status for all the LNs was established through correlation with pathology reports. Similarly, the correlation of the contoured lymph nodes with the pathology report was initially performed by the research scientist (S.R.) and then verified and edited by the senior radiation oncologist (D.S.). Each contoured lymph node was matched to its corresponding pathology entry by nodal level (I–VI) and anatomical side as described in the reports. Malignant nodes were identified and labeled based on explicit positive findings in the surgical pathology reports. Correspondingly, benign‐node labeling also relied on pathology documentation. Patients were included only if their pathology reports explicitly documented the location, number, and laterality of malignant and benign lymph nodes. Ambiguous cases were reviewed by a senior radiation oncologist and excluded if uncertainty persisted. As the correlation between the pathology report and imaging involved a degree of subjective judgment, only cases with a high degree of confidence were retained. An example of the primary tumor and the lymph nodes on the CT scan is shown in Figure 1.

### 2.2 Model workflow

**FIGURE 2:**
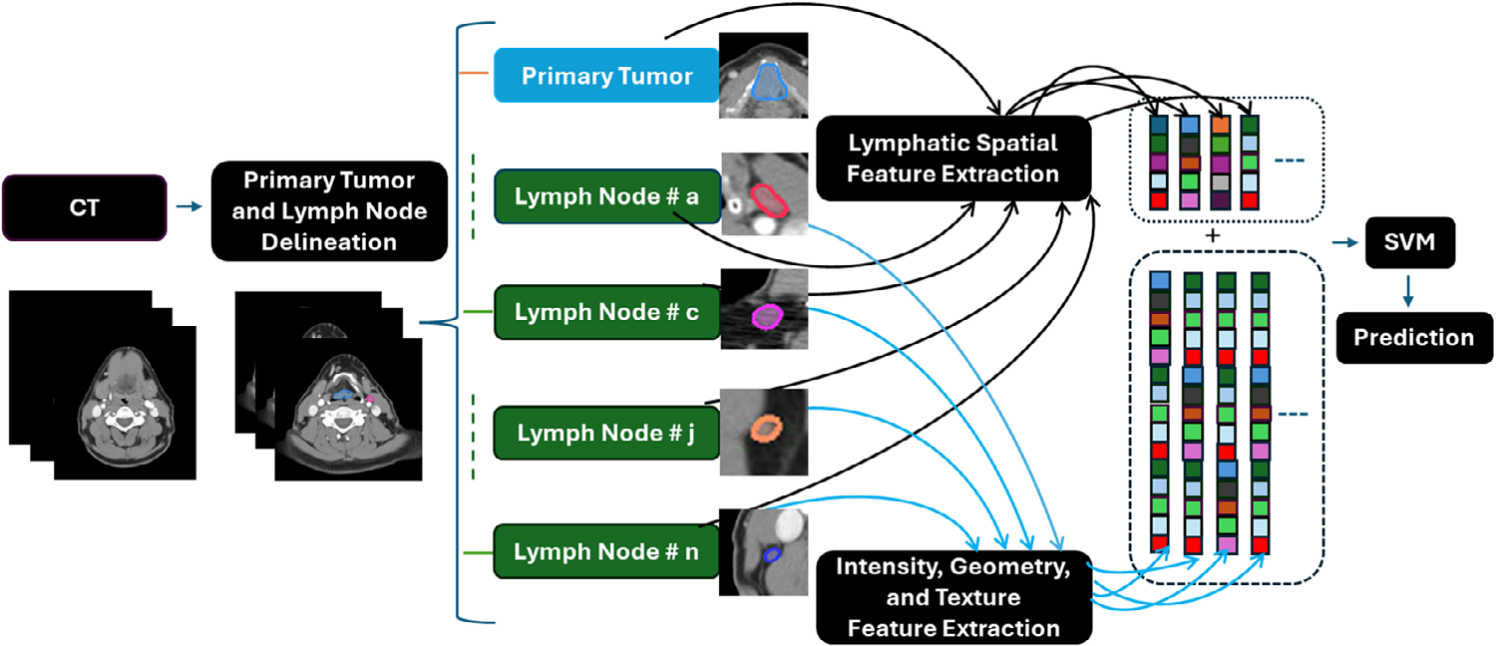
Workflow of the proposed spatially aware radiomics model.

The workflow for the proposed spatially aware radiomics model is depicted in Figure 2. It involves the following steps: (i) retrieve the CT scan; (ii) contour the primary tumor and the lymph nodes; (iii) extract the conventional radiomics features including intensity, geometry, and texture features; (iv) derive the lymphatic spatial features via calculation of the distance between the lymph node and primary tumor, consideration of location of the primary tumor, identification of the lymph node level, and determination of the laterality of both the lymph node and the primary tumor; (v) establish the Gaussian‐kernel‐based support vector machine (SVM) model to predict the malignancy of the lymph node. This proposed workflow incorporates the spatial relationship between the node and the primary tumor, and the malignancy involvement influenced by different primary tumor types, representing a more comprehensive analysis than conventional radiomics models that rely solely on imaging features.

### 2.3 Feature extraction

Conventional radiomics features, including intensity, geometry, and texture‐based descriptors, were extracted for each contoured lymph node on CT images. A total of 56 conventional radiomics features were extracted. Nine intensity features included minimum, maximum, mean, median, standard deviation, sum, skewness, kurtosis, and variance. Seven geometry features included volume, major diameter, minor diameter, eccentricity, elongation orientation, bounding box volume, and perimeter. For texture analysis, we employed a comprehensive approach beyond the conventional Gray‐Level Co‐occurrence Matrix (GLCM), incorporating four gray‐level‐based matrices: GLCM, Gray‐Level Run Length Matrix (GLRLM), Gray‐Level Size Zone Matrix (GLSZM), and Neighboring Gray‐Tone Difference Matrix (NGTDM). GLCM captures spatial relationships and texture patterns within the contoured lymph node^10^; GLRLM evaluates the length of homogeneous runs of pixels, reflecting texture roughness^11^; GLSZM measures the size distribution of homogeneous zones^12^; and NGTDM quantifies local intensity variation, which is sensitive to subtle texture changes.^13^ This multi‐faceted methodology enables robust and detailed characterization of tissue heterogeneity compared to using GLCM alone.

Furthermore, five additional spatially and anatomically relevant features were incorporated:

1. Primary tumor type
2. Lymph node level
3. Laterality of the lymph node
4. Laterality of the primary tumor
5. Distance from the lymph node to the primary tumor

**FIGURE 3:**
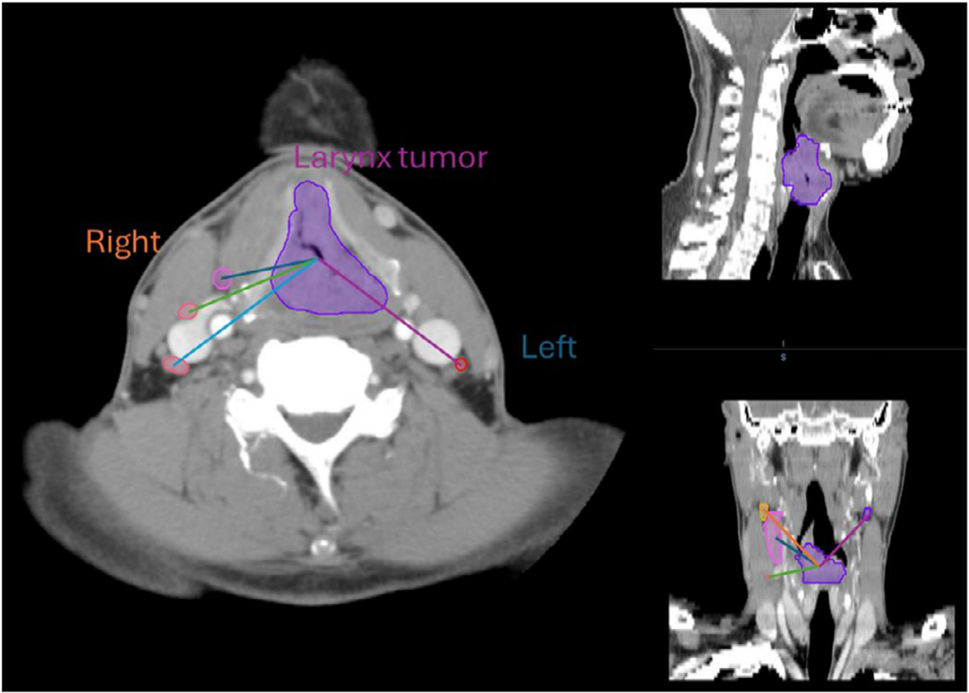
An example of distance between the primary tumor and the lymph nodes.

The primary tumor type here includes tonsil, base of tongue, oropharynx (crossing several sites), larynx, and hypopharynx. These features were derived using a combination of image metadata, anatomical landmarks, and clinical annotations. For features (1)–(4), the numerical value assignment is listed in Table 1. While these encodings imply an arbitrary ordinal relationship, the nonlinear mapping provided by the Gaussian‐kernel‐based SVM helps the model treat these more like nominal categories. Future work will explore the use of one‐hot encoding for these nominal features to strictly avoid any unintended ordinal bias. To calculate the distance from each node to the primary tumor, the centroid locations of the lymph node and the primary tumor are firstly detected, and then the Euclidean distance between them (as shown in Figure 3) was derived.

**TABLE 1:** Numerical value assignment for the lymphatic spatial features (1)–(4).

Features	Name	Numerical value
Primary tumor type	Base of Tongue	1
	Tonsil	2
	Larynx	3
	Hypopharynx	4
	Oropharynx	5
Lymph node level	I	1
	II (IIa, IIb)	2
	III	3
	IV	4
	V (Va, Vb)	5
	VI	6
LN laterality	Left	0
	Right	1
Primary tumor laterality	Left	0
	Right	1
	NA	2

### 2.4 Model development and evaluation

A predictive model was constructed using a Gaussian‐kernel‐based SVM algorithm. The Gaussian kernel was selected due to its capacity to model the nonlinear relationships inherent in radiomics features. To solve the optimization problem, the method of Lagrange multipliers was applied to reformulate the primal problem, and the Sequential Minimal Optimization (SMO) algorithm was employed to identify the optimal solution.

Two models were developed: a baseline SVM only using 56 conventional radiomics features and an enhanced model using both the 56 conventional radiomics features and 5 additional spatial/anatomical features. The development of these two distinct models was designed specifically to demonstrate the value of integrating anatomical knowledge and clinical factors compared to solely relying on lymph node features for malignancy prediction. To evaluate the model performance, we employed a five‐fold nested cross‐validation strategy. All data preparation steps including normalization and imbalance handling were performed independently within each iteration of the cross‐validation. Specifically, for each iteration, Min‐Max scaling factors for feature normalization were derived solely from the training set and applied consistently to the corresponding independent testing set. Synthetic Minority Over‐sampling Technique (SMOTE) was only utilized in the training stage to address the class imbalance issue.

1. Outer loop (Testing): The patient cohort was partitioned into five nonoverlapping folds on a per‐patient basis to prevent data leakage. For each iteration, one‐fold served as the independent test set for final, unbiased evaluation.
2. Data preparation and imbalance handling (within iteration):
3. Min‐Max Scaling: All 61 features were normalized using Min‐Max scaling (to the range of [0, 1]). The scaling factors (minimum and maximum values) were derived only from the training data of that iteration and applied consistently to normalize the corresponding independent test set features.
4. SMOTE application: to address the severe class imbalance (1119 benign vs. 270 malignant nodes). SMOTE was applied to the training data to generate synthetic samples for the malignant class, creating a balanced dataset for classifier training.
5. Inner loop (Hyperparameter Tuning): The remaining four folds were used in an inner cross‐validation procedure to systematically tune the key hyperparameters, including the box constraint (C) and the kernel parameter (σ). The set of hyperparameters yielding the highest classification accuracy on the inner validation sets was selected as optimal.

The final model for each iteration was trained with the optimal hyperparameters selected from the inner loop and evaluated on the independent outer test set. The model performance was assessed using sensitivity (SEN), specificity (SPE), accuracy (ACC), the area under the receiver operating characteristic curve (AUC), the positive predictive value (PPV) and the negative predictive value (NPV). The statistical comparisons of model predictions were conducted using a paired t‐test in accuracy and a DeLong test^14^ in AUC (*p* < 0.05 considered significant), respectively.

## 3 RESULTS

The baseline radiomics model, developed using conventional intensity, geometry, and texture features, achieved the following performance: SEN of 0.915 (95% CI: 0.900‐0.930), SPE of 0.756 (95% CI: 0.733‐0.779), ACC of 0.787 (95% CI: 0.765‐0.808), and AUC of 0.931 (95% CI: 0.913‐0.947). In contrast, the proposed spatially aware radiomics model, which integrated anatomical knowledge and spatial features, demonstrated significant performance improvements: SEN of 0.919 (95% CI: 0.904‐0.933), SPE of 0.845 (95% CI: 0.826‐0.864), ACC of 0.860 (95% CI: 0.841‐0.878), and AUC of 0.953 (95% CI: 0.938‐0.966) (Table 2). Note that, exact 95% confidence intervals for ACC, SEN, and SPE were computed using the Clopper–Pearson method based on the binomial distribution, providing conservative coverage for proportions. The 95% confidence interval (CI) for the AUC was estimated using the bias‐corrected and accelerated (BCa) bootstrap method with 1000 resamples, which accounts for both bias and skewness in the bootstrap distribution. This approach provides an accurate, nonparametric estimate of uncertainty for ROC‐based metrics even in moderately imbalanced datasets.

**TABLE 2:** Performance comparison between models.

Model	Sensitivity	Specificity	Accuracy	AUC	PPV	NPV
Baseline Radiomics	0.915	0.756	0.787	0.931	0.475	0.974
Spatially Aware Radiomics	0.919	0.845	0.860	0.953	0.589	0.977

Further evaluation revealed that the enhanced model achieved a PPV of 0.589 and a NPV of 0.977, compared to 0.475 (PPV) and 0.974 (NPV) for the baseline model. These results indicate a notable gain in the model's ability to correctly identify malignant lymph nodes (PPV: +11.4%) without sacrificing the model's reliability in ruling out benign nodes (NPV: +0.3%).

Statistical testing using a paired t‐test confirmed that the improvements in accuracy achieved by the spatially aware model were statistically significant (*p* = 3.71 × 10^−^ ^20^). To further assess whether the improvement in discrimination was significant, we applied DeLong's nonparametric test^14^ for AUCs, which compares ROC curves built on the same cases via U‐statistics‐based variance estimate, and found a significant difference (*p* = 1.13 × 10^−^^4^), confirming superior AUC for the spatially aware model.

**FIGURE 4:**
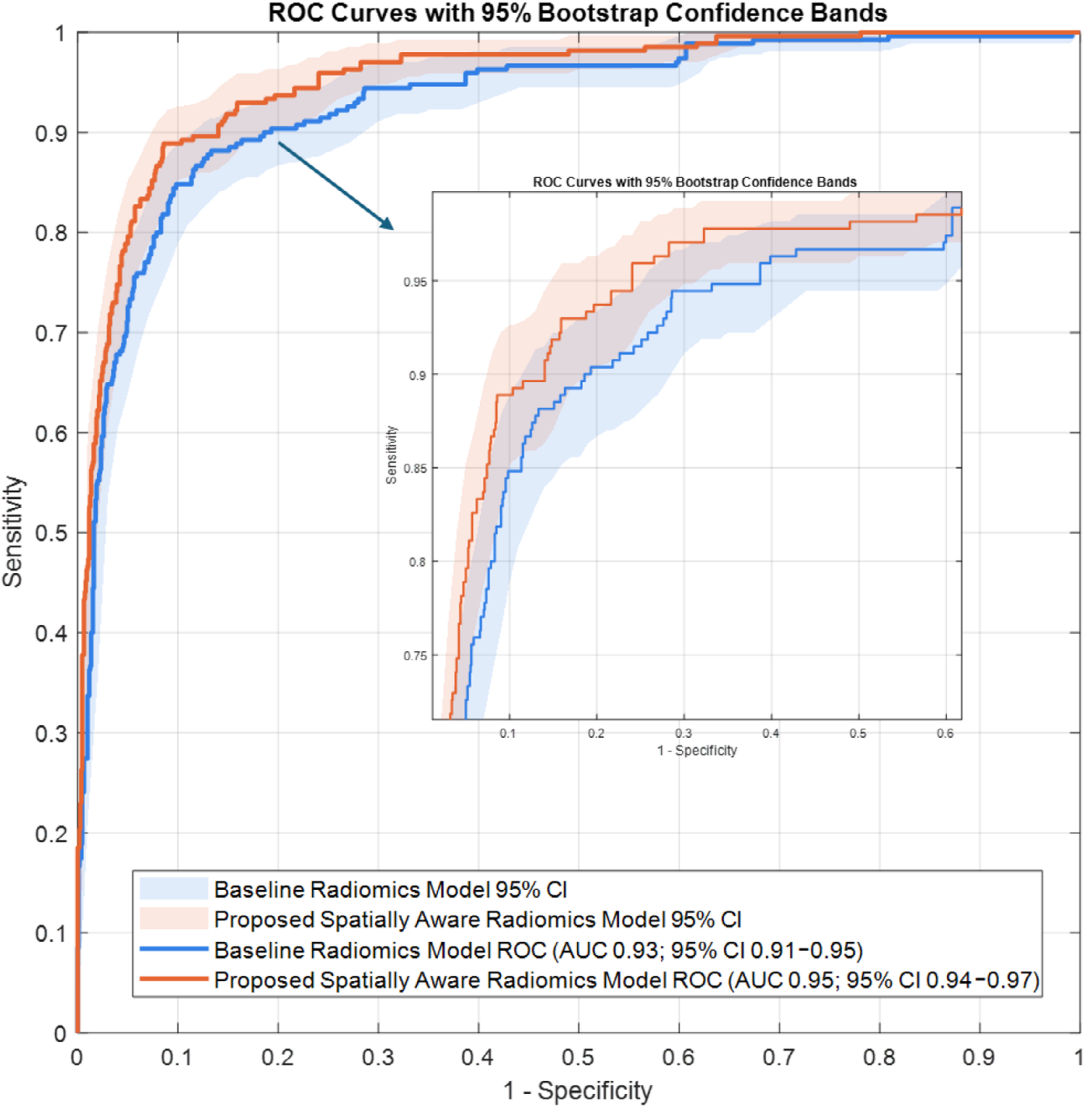
The ROC curves with 95% bootstrap confidence bands of the baseline radiomics model and the proposed spatially aware radiomics model.

The ROC curves (Figure 4) demonstrate superior classification performance by the proposed model, particularly in the low‐false‐positive region, highlighting its enhanced utility in clinical settings where over‐treatment is a concern.

## 4 DISCUSSION

Radiation therapy is one of the most common treatment modalities for HNC.^15^, ^16^, ^17^ In preparation for treatment, a CT simulation is typically performed using immobilization devices to ensure consistent and reproducible patient positioning throughout the course of radiotherapy. While PET/CT can support nodal malignancy identification, using it as a planning reference depends on image registration with the simulation CT. Multiple registration steps may be required, and the associated uncertainty could impact clinical decision‐making. To mitigate such limitations, we focus on developing a malignancy prediction model that relies solely on simulation CT, which is already integrated into the standard radiation therapy workflow, bypassing the need for PET/CT fusion or additional imaging that can introduce registration uncertainties or delay treatment planning. This efficiency aligns with broader efforts to streamline care and reduce dependency on resource‐intensive modalities.

Recent studies have begun to explore spatially aware radiomic approaches. For instance, Bae et al.^18^ showed that integrating spatial information from pretreatment MRI can model the development of distant brain metastases in patients undergoing stereotactic radiosurgery. These findings suggest that spatially aware radiomics can provide valuable insights into tumor behavior and metastasis patterns.​ The proposed study highlights the value of incorporating anatomical knowledge and spatial context into radiomics models for lymph node malignancy prediction in HNC. The proposed spatially aware model outperformed the conventional approach in all key metrics, including sensitivity, specificity, and overall accuracy. Notably, the 8.9% gain in specificity and 11.4% increase in PPV underscore its potential clinical impact, improving confidence in identifying true malignant nodes while reducing the risk of false positives.

In head and neck radiotherapy, overtreatment of benign lymph nodes carries risks of increased toxicity to critical surrounding structures, such as salivary glands, spinal cord, and swallowing muscles. Traditional radiomic (and clinical) classification models often rely heavily on size and texture cues, which may be insufficient in small or borderline lymph nodes. By integrating lymphatic spread patterns (e.g., laterality, lymph node level) and tumor‐specific anatomical information (e.g., distance to primary tumor), our model aligns more closely with how expert clinicians evaluate nodal status.

The model's improved metrics also offer direct clinical decision support. The high NPV of 0.977 from the spatially aware model ensures that the model is reliable in excluding benign nodes from the elective radiation, thereby reducing treatment‐related toxicity to nearby critical structures.^19^, ^20^, ^21^ Whereas the observed increase in PPV from 0.475 (baseline radiomics model) to 0.589 means that the spatially aware model offers better predictive confidence when flagging a node as malignant, further work is still needed to reduce the false positive rate. Currently, the reported results are derived based on a classification threshold of 0.5. In clinical practice, the selection of this operating point can be strategically adjusted to align with specific clinical intents. For instance, a higher decision threshold may be selected to prioritize the sparing of benign nodes from unnecessary elective irradiation (high specificity), whereas a lower threshold could be utilized in high‐risk cases to prioritize the detection of all potential malignancies and minimize the risk of missed disease (high sensitivity). The integration of expert‐derived anatomical features into a machine learning pipeline also supports interpretability, an important factor in clinician trust and clinical adoption. Each spatial feature (e.g., lymph node level, laterality) has a clear physiological rationale grounded in established lymphatic drainage patterns, enhancing the explainability of model predictions.

One limitation of this study is the inherent uncertainty in correlating individual lymph nodes on CT imaging with their corresponding pathological findings. Although lymph nodes were matched by anatomical level and laterality and only cases with detailed surgical and pathology documentation were included, one‐to‐one node correspondence cannot be guaranteed with absolute certainty. To minimize ambiguity, all questionable cases were reviewed by a senior radiation oncologist, and nodes with uncertain mapping were excluded. These steps improve label reliability but also reflect an unavoidable constraint when using retrospective surgical pathology as the reference standard. This process may also introduce bias by preferentially retaining lymph nodes with more identifiable characteristics on imaging, such as larger size or necrotic appearance, potentially influencing the model to learn known correlates of malignancy rather than subtler radiomic patterns. Beyond pathological correspondence, several additional limitations should be acknowledged. First, this study was conducted retrospectively at a single institution, which may limit generalizability to other clinical settings with different imaging protocols, contouring guidelines, or patient populations. Second, while the current model focuses on features that provide node‐specific risk context (such as distance and nodal level), primary tumor‐specific features like tumor volume, T‐stage, or primary tumor radiomics could capture overall disease aggressiveness. Future work could explore the optimal strategy for incorporating such patient‐level features to further enhance the model's predictive capability without sacrificing node‐specific resolution. Third, although class imbalance mitigation strategies were applied, the dataset contained substantially more benign than malignant lymph nodes, which could influence model behavior despite cross‐validation. Fourth, all contours were generated or supervised by experienced head and neck specialists, which strengthens consistency but may not reflect real‐world contouring variability across observers or institutions. Finally, the model has not yet undergone external validation; multicenter testing will be essential to assess robustness and clinical utility prior to deployment.

Future directions will include a comparative analysis of the proposed model against other advanced machine learning classifiers, such as random forests and neural networks, to confirm the optimal architecture for this prediction task. External validation using multi‐institutional datasets to assess generalizability, and prospective studies evaluating the impact of this tool in clinical decision‐making workflows are also planned. Integration into radiation treatment planning systems could support adaptive nodal contouring, potentially leading to more personalized and precise radiotherapy plans.

## 5 CONCLUSION

This study introduces a spatially aware radiomics model that integrates anatomical and spatial context to improve lymph node malignancy prediction in HNC. The model significantly enhances predictive performance, particularly in terms of specificity and PPV, without sacrificing sensitivity or NPV. By embedding clinical reasoning into the model design and relying solely on simulation CT, this approach provides an interpretable and workflow‐compatible decision‐support tool. Future work will focus on external validation and clinical integration into radiation treatment planning systems.

## AUTHOR CONTRIBUTIONS

**Liyuan Chen**: study conception, data curation, model development, and manuscript writing; **Sepeadeh Radpour**: data collection, preprocessing, and radiomics feature extraction; **Michael Dohopolski**: data preparation, and clinical data interpretation; **David Sher**: clinical guidance, interpretation of results, and manuscript review; **Jing Wang**: project supervision, study design, and critical manuscript revision.

## CONFLICT OF INTEREST STATEMENT

The authors declare no conflicts of interest.
